# Research progress on the activation mechanism of NLRP3 inflammasome in septic cardiomyopathy

**DOI:** 10.1002/iid3.1039

**Published:** 2023-10-04

**Authors:** Yuqi Wen, Yang Liu, Weihong Liu, Wenli Liu, Jinyan Dong, Qingkuo Liu, Hao Hao, Hongsheng Ren

**Affiliations:** ^1^ Shandong University of Traditional Chinese Medicine Jinan China; ^2^ Affiliated Hospital of Shandong University of Traditional Chinese Medicine Jinan China; ^3^ Department of Intensive Care Unit Shandong Provincial Hospital Affiliated to Shandong First Medical University Jinan China

**Keywords:** activation mechanism, cytokines, inflammatory response, nucleotide‐binding oligomerized structural domain‐like receptor protein 3 inflammasome, septic cardiomyopathy

## Abstract

Sepsis is an uncontrolled host response to infection, resulting in a clinical syndrome involving multiple organ dysfunctions. Cardiac damage is the most common organ damage in sepsis. Uncontrolled inflammatory response is an important mechanism in the pathogenesis of septic cardiomyopathy (SCM). NLRP3 inflammasome promotes inflammatory response by controlling the activation of caspase‐1 and the release of pro‐inflammatory cytokines interleukin IL‐1β and IL‐18. The role of NLRP3 inflammasome has received increasing attention, but its activation mechanism and regulation of inflammation in SCM remain to be investigated.

## Graphical abstract

Inflammasomes play a key role in the development and progression of innate immunity and immunoinflammatory diseases. It was found that NLPR3 inflammasomes are closely related to sepsis and septic cardiomyopathy (SCM) and can be involved in regulating SCM through cardiomyocyte inflammatory response and apoptosis, oxidative stress injury, calcium regulation, endoplasmic reticulum stress, mitochondrial dysfunction, and exosomes.

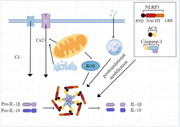

## 1 INTRODUCTION

Sepsis is a systemic inflammatory syndrome caused by infection, which can lead to multiple organ failure and shock due to an overreacted immune response.^1^, ^2^ It is one of the most common critical diseases in the clinic, usually life‐threatening. Septic cardiomyopathy (SCM) is a form of sepsis with myocardial damage. It is an acute syndrome of cardiac insufficiency based on systemic infection and inflammation, which can accelerate the failure of the cardiovascular system, and worsen microcirculation disturbance and hypoperfusion in patients.^3^, ^4^, ^5^ The prevalence of SCM in patients with sepsis is 10%–70%.^4^ There are no definitive diagnostic criteria for SCM. Most review articles and experts generally agree that the main manifestations of SCM are: (1) left ventricular dilatation, normal or reduced filling pressure; (2) decreased ventricular contractility; (3) right ventricular (systolic or diastolic) dysfunction or left ventricular dysfunction, decreased response to volume infusion.^5^, ^6^ The occurrence and development of SCM are related to the immune system and cardiovascular system.^7^ The pathological mechanisms are complex and diverse, including activation of the inflammatory response,^8^ dysregulation of calcium ion regulation, mitochondrial dysfunction,^9^ and oxidative stress.^10^ When the host encounters damage, infection, or virus, the intrinsic pathogen associated molecular patterns (PAMPs) and damage‐associated molecular patterns (DAMPs) are activated. After recognizing specific pathogen structures through pattern recognition receptors (PRRs), the body releases a large number of inflammatory mediators, leading to inflammatory cascade reactions. Inflammation generates corresponding immune responses, clearing pathogens and resisting external invasion.^11^ When the body is infected by various pathogens, it will lead to a series of immune responses. The body activates the mononuclear macrophage system (neutrophils, macrophages, etc.) through endogenous ligands, increases the activity of nuclear transcription factors, and promotes the synthesis and release of cytokines and complement (such as IL‐6, IL‐1, TNF‐α, and C5a). At the same time, the body will have abnormal coagulation function, resulting in direct myocardial cell damage and myocardial tissue hypoperfusion.^12^ The interactions between cells in the innate immune system cannot be ignored. Macrophages can produce chemokines and recruit monocytes and neutrophils to the infected site. Neutrophils, as important sources of cytokines and chemokines, can also activate and recruit other cells in the immune system. In addition to activating the functions of other innate cells, neutrophils can also suppress immune responses and promote inflammation regression. It can promote antibacterial and epidemic prevention mechanisms by phagocytosis, releasing soluble antibacterial agents from its particles, and releasing neutrophil extracellular traps (NET).^13^ Nucleotide‐binding oligomerization domain‐like receptor protein3 (NLRP3) is a cytoplasmic immune factor that responds to cellular stress signals. It is usually activated in response to infection or inflammation in the host, forming NLRP3 inflammasomes and inducing apoptosis.^14^ NLRP3 is involved in the occurrence and development of a variety of diseases such as inflammation, immunity, and metabolism. It is an important innate immune sensor that can respond to various DAMPs and induce inflammation.^15^ It has been shown that NLRP3 inhibition can improve cardiac function and increase survival. In a murine model of sepsis by cecum ligation and puncture (CLP) and in lipopolysaccharide（LPS）‐induced cardiac fibroblasts, corticosteroid pretreatment was found to inhibit NLRP3 formation, cysteine asparaginase‐1 activation, and IL‐1β secretion. This demonstrates that cortisone is a novel immunomodulatory factor with the ability to inactivate NLRP3 inflammasomes and protect the myocardium from septic injury.^16^ Ulinastatin (UTI) inhibited the activation of NLRP3 inflammasomes, and high doses of UTI significantly preserved myocardium and improved survival in septic rats.^17^ NLRP3 knockout mice have milder symptoms of SCM.^18^ There is increasing evidence that NLRP3 inflammasome overactivation is associated with a wide range of inflammatory diseases, but the mechanism of NLRP3 inflammasome activation and its regulation of inflammation in SCM remains to be investigated. This review describes the function of NLRP3 and its activation mechanism in SCM. Further study of this mechanism may provide a basis for reducing inflammation, increasing tissue blood perfusion, improving cardiac function, and finding new therapeutic targets in patients with SCM.

## 2 NLRP3 INFLAMMASOME AND ITS ACTIVATION MECHANISM

Innate immunity is known as the first line of defense against pathogenic infections, injuries, and stress‐induced damage, and is induced by PRRs.^19^ Among the different PRRs families identified so far are Toll‐like receptors (TLRs), NOD‐like receptors (NLRs), C‐type lectin receptors (CLRs), melanoma 2 (AIM2)‐like receptors (ALRs) deletions, retinoic acid‐inducible gene 1 (RIG‐I)‐like receptors (RLRs), cyclic GMP‐AMP synthase (cGAS)‐trunk Perturbin gene stimulator (STING), and Pyrin. Among these PRRs, the NLRs are the largest and most diverse family, with 22 NLRs having been identified in humans. Structurally, these family members share similar domains: an N‐terminal effector domain, a central nucleotide‐binding domain (NBD/NOD/NACHT), and a C‐terminal leucine‐rich repeat (LRR). The NLR inflammasome is a polyprotein oligomer composed of upstream sensor proteins (Nod‐like receptors), adaptor proteins, apoptosis‐related spot‐like proteins (including CARD(ASC)), and downstream effector proteins caspase‐1.^20^, ^21^ The activation of inflammatory bodies promotes the activation of caspase‐1 and the maturation and secretion of IL‐18 and IL‐1β. IL‐1 β and IL‐18 play an important role in immune response and inflammation, such as promoting the recruitment of neutrophils and inducing the differentiation of Th17 cells. Therefore, inflammasomes are not only crucial in the body's anti‐infective immunity, but also participate in the occurrence and development of various major inflammatory‐related diseases.^22^ So far, a variety of NLR inflammasomes have been described, among which NLRP3 inflammasomes are the most extensively studied. When NLRP3 inflammasome assembly is activated, the N‐terminal pyrin domain (PYD) of the NLRP3 protein interacts with the PYD domain of the ACS, which links the receptor protein to the effect protein by recruiting pro‐caspase‐1. The effector protein caspase‐1 shears the inactive inflammatory factors pro‐IL‐18 and pro‐IL‐1β into mature IL‐18 and IL‐1β, and also proteolytically hydrolyzes gasdermin D (GSDMD) to form the GSDMD pore, which leads to inflammation and cell necrosis.^23^

**Figure 1:**
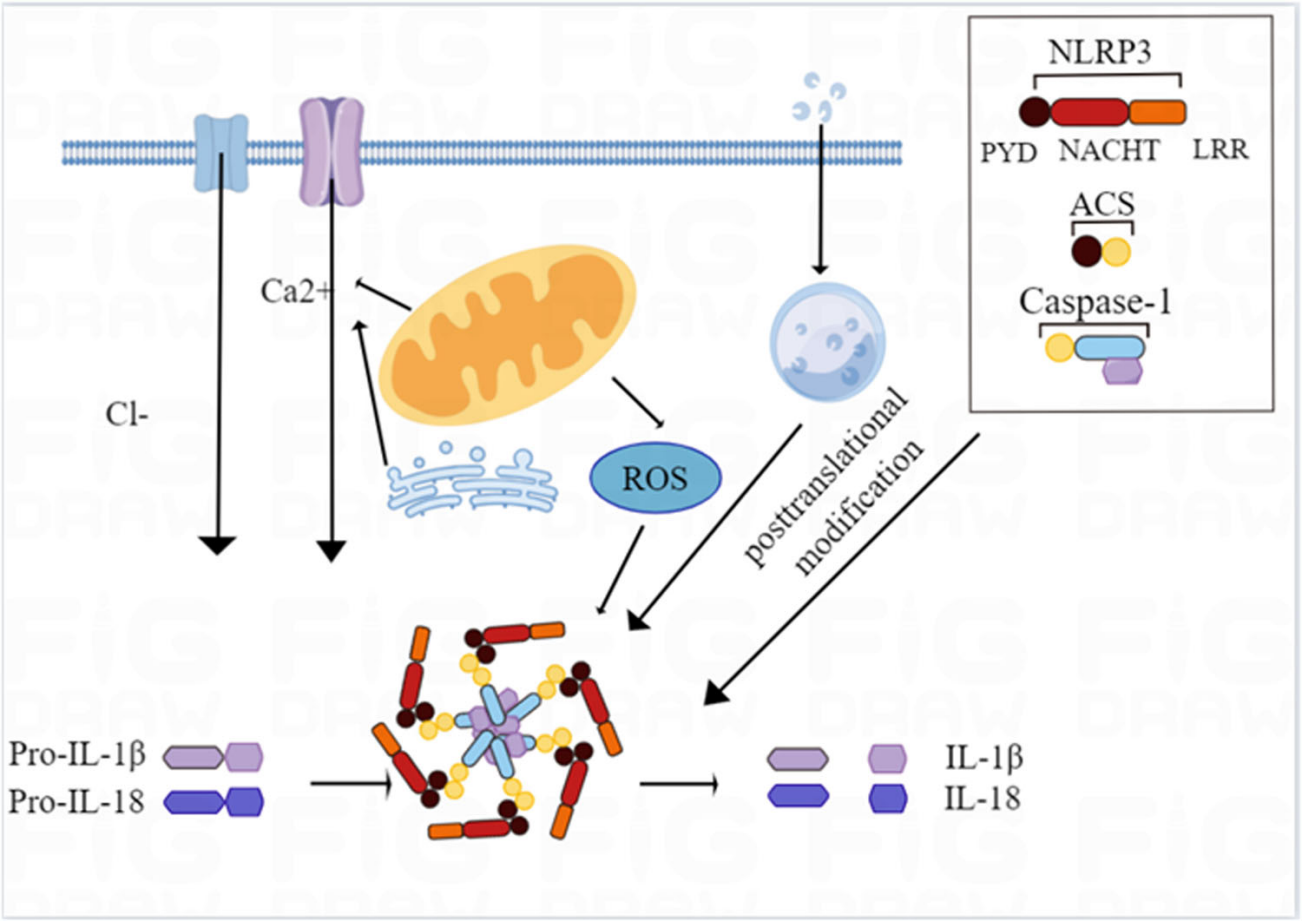
Mechanism of inflammasome activation.

The factors and molecular mechanisms of NLRP3 inflammasome activation are currently thought to be divided into three main pathways: the first pathway is the opening of extracellular ATP‐stimulation‐dependent ion channels, which directly promotes the aggregation and activation of NLRP3 inflammasome by promoting K^+^ efflux and the formation of connexin membrane channels.^24^ The second pathway is the endocytosis of extracellular crystals or specific particles into cells, causing a lysosomal rupture and promoting the aggregation and activation of NLRP3 inflammasome.^25^ The third pathway is that PAMPs and DAMPs promote increased intracellular ROS production through ROS‐dependent signaling pathways and promote aggregation and activation of NLRP3 inflammasome (Figure 1). In addition, NLRP3 posttranslational modifications such as phosphorylation and ubiquitination, as well as individual uptake and metabolic processes, also promote the activation of inflammasomes. In conclusion, the activation mechanism of the NLRP3 inflammasome is complex, incorporating many pathways and processes that promote inflammation.^26^

## 3 ACTIVATION MECHANISM OF NLRP3 INFLAMMASOME ASSOCIATED WITH SCM

### 3.1 Inflammatory response

Potential causes of SCM include PAMPs, such as LPS, lipid cholic acid, cytokines and nitric oxide, and bacterial DNA. These mediators bind to pattern recognition receptors. Interactions between PRRS and PAMPs can activate intracellular signal transduction pathways. This leads to the activation of nuclear factor‐κB (NF‐κB) and p38/MAPK signaling pathways that trigger an inflammatory cascade that produces numerous inflammatory factors such as TNF‐α, IL‐6, IL‐8, and IL‐1β.^13^, ^27^ It has been clearly shown that the maturation and activation of IL‐1β are regulated by caspase‐1, and the regulation of caspase‐1 is closely related to the NLRP3 inflammasome. NLPRP3 inflammasome promotes inflammation by controlling caspase‐1 activation in macrophages and the release of IL‐1β and IL‐18.^21^ Busch et al.^28^ showed that in NLRP3 knockout mice, sepsis failed to reactivate NLRP3 inflammasome, IL‐1β production was greatly reduced, and activation of NF‐κB and NF‐κB signaling was diminished. Zhang et al.^29^ found that inhibiting NLRP3 inflammasome activation in cardiac fibroblasts could attenuate LPS‐induced myocardial dysfunction in mice and improve survival in septic mice. In addition, Zhang W showed that carbon monoxide‐releasing molecules inhibit the activation of NLRP3 inflammasome by blocking the interaction between NLRP3 inflammasome and adaptive protein ASC, thus alleviating myocardial dysfunction in sepsis mice.^30^ And sulfur dioxide can downregulate NLRP3 expression in septic rats through the TLR4/NLRP3 signaling pathway, which in turn interferes with its downstream inflammatory cytokines and attenuates cardiac insufficiency.^31^ The above findings suggest that the development of SCM may be associated with an excessive inflammatory response, but extensive studies are still needed to further clarify the important mechanisms of NLRP3 in the inflammatory activation of SCM.

### 3.2 Oxidative stress injury

Reactive oxygen species (ROS) and reactive nitrogen species (RNS) were significantly increased in myocardial cells during sepsis.^32^ Free radicals are a class of unstable and highly oxidizing molecules. Oxygen‐containing free radical molecules formed in biological systems and their predecessors are called ROS, including superoxide anion, hydrogen peroxide, and hydroxyl radical. RNS mainly includes peroxynitrite formed by nitric oxide and oxygen free radicals. Excess ROS disrupt cell signaling, activate inflammatory factors, and induce lipid peroxidation. This even leads to cell death, which induces cellular damage.^33^ In heart disease, ROS is considered to be one of the key factors in the activation of NLRP3.^34^ The study found that NLRP3 inflammasome activation increased significantly in a ROS‐dependent manner, inducing cell death under LPS stimulation. Inhibition of ROS production by NAC decreased the activation of NLRP3 inflammasome and alleviated LPS‐induced H9C2 cell damage.^35^ The mechanisms by which ROS‐activated NLRP3 are not fully understood, but studies have shown that thioredoxin interacting Proteins (TXNIP), one of the ligands of NLRP3 inflammasomes, were sensitive to ROS.^36^ In the basal state, TXNIP binds thioredoxin‐reducing protein and inhibits the antioxidant activity of the thioredoxin‐reducing protein. When ROS concentration increases, TNXIP separates from thioredoxin and then interacts with the NACHT domain of NLRP3 inflammasomes to oligomerize NLRP3 protein and promote inflammasome activation.^37^, ^38^ Rahim demonstrated that melatonin improves the prognosis of SCM by inhibiting the NLRP3/Nrf2 antioxidant pathway induced by ROS.^39^ The study of ROS‐mediated activation and regulation mechanism of NLRP3 inflammasome in sepsis will provide theoretical guidance as well as new therapeutic targets for the treatment of sepsis‐induced cardiac dysfunction.

### 3.3 Mitochondrial dysfunction

Myocardial mitochondrial damage or dysfunction induced by sepsis is an important cause of myocardial cell metabolic disorders, oxidative stress, immune dysregulation, and insufficient energy production, causing myocardial cell dysfunction and eventually heart failure and even death. Causes of mitochondrial dysfunction in SCM include activation of oxidative stress, increased mitochondrial membrane permeability, mitochondrial uncoupling, mitochondrial energy metabolism disorders, mitochondrial autophagy, and so on.^40^, ^41^, ^42^ Mitochondria are the main source of production of mitochondrial reactive oxygen species (mtROS). Excessive accumulation of ROS activates toll‐like receptor‐mediated inflammatory pathways and exacerbates myocardial injury in sepsis.^43^ ROS accumulation can also oxidatively modify macromolecular protein and DNA structures and disrupt the mitochondrial structure, increase mitochondrial membrane permeability, and activate apoptotic pathways by cytochrome C escape outside the cell, leading to myocardial cell apoptosis.^44^ Studies have shown that during cell stress, the level of mtROS increases significantly, which can activate NLRP3 inflammasome after entering the cytoplasm.^45^

Mitochondrial autophagy removes damaged, abnormally functioning mitochondria from releasing apoptotic signals that cause apoptosis.^46^ When mitochondria receive external stimuli, mitochondrial autophagy PENT induces activation of the kinase1/Parkin pathway, which mediates mitochondrial autophagy to occur to complete the clearance of damaged mitochondria, thereby reducing ROS, mtDNA levels, and inhibiting NLRP3 inflammasomes^47^ and the use of mitochondrial autophagy inhibitors can contribute to NLRP3 inflammasome activation.^48^ Imiquimod is a small molecule adenine derivative that induces mtROS, activates NLRP3 inflammasome by inhibiting quinone oxidoreductase and mitochondrial complex Ⅰ, and participates in the inflammatory response.^49^ The main regulators of mitochondrial biosynthesis are considered to be the nuclear gene peroxisome proliferator‐activated receptor‐γcoactivator‐1α (PGC‐1α) and nuclear factor erythroid‐derived 2‐like 2 (Nrf2).^50^ Nrf2 is a major regulator of the body's antioxidant defense system. On the one hand, Nrf2 can reduce ROS production by inducing the expression of antioxidant‐related genes, thereby inhibiting NLRP3 activation.^51^ On the other hand, it reduces NLRP3 inflammasome activity by inhibiting NF‐κB activation and decreasing the expression of caspase‐1, IL‐1β, and IL‐18. In the cecum ligation perforation sepsis model, PGC‐1α and Nrf2 were both elevated at the transcriptional level after exogenous sodium thioredoxin treatment in a dose‐dependent manner and could promote mitochondrial biosynthesis by promoting the expression of mitochondrial transcription factor A (a mitochondrial protein encoded by a cytosolic gene), which in turn improved mitochondrial energy metabolic function and attenuated myocardial injury.^52^ The mtDNA released during mitochondrial injury acts as a DAMP and can be co‐immunoprecipitated with NLRP3 specifically activating NLRP3 inflammasome.^53^ When mitochondria are destabilized, cardiolipin redistributes to the outer surface of mitochondria and activates NLRP3 inflammasome by directly binding to the LRR structure domain of NLRP3.^54^ In addition, the formation of NLRP3 inflammasome assembly requires the involvement of multiple mitochondrial proteins such as mitochondrial antiviral signaling protein (MAVS) and mitochondrial fusion protein 2 (Mfn2).^55^, ^56^ Therefore, the mitochondria not only release the MAVS but also the mitochondrial fusion protein (Mfn2). Therefore, mitochondria not only release ROS, mtDNA, and cardiolipin to directly activate NLRP3 inflammasome but also recruit NLRP3 and promote NLRP3 inflammasome assembly through MAVS and Mfn2. Exploring the mechanism of mitochondrial regulation of NLRP3 inflammasome activation in cardiomyocytes during sepsis leading to myocardial injury and the method of restoring mitochondrial function may provide new ideas for the clinical treatment of myocardial injury in sepsis.

### 3.4 Exosomes

With the development of molecular biology technology, people are discovering new ways and model factors to regulate cell biological functions. Exosomes (30–150 nm in diameter) are produced from multivesicular bodies (MVBs) membranes and can deliver a variety of biomolecules that regulate intercellular communication under normal and pathophysiological conditions.^57^ According to Vesiclepedia, a total of 349,988 proteins, 639 lipids, 27,646 messenger RNAs (mRNAs), and 10,520 microRNAs (miRNAs) have been identified in exosomes. In recent years, exosomes have been key signaling molecular carriers in the inflammatory process of transferring proteins, lipids, and nucleic acids, thereby affecting the metabolism of target cells in many diseases, including cancer, cardiovascular disease, and neurodegenerative diseases.^58^ Exosomes are also thought to play an important role in SCM. The results showed that miR‐223 negatively regulated the expression of NLRP3 and downregulated the expression levels of Capsase‐1 and IL‐1β to enhance the viability of cardiomyocytes.^59^ miR‐129‐5p alleviates cardiomyocyte injury by targeting TRPM7 and inhibiting NLRP3 inflammasome activation.^60^ miR‐484 inhibitors significantly increased LPS‐treated cardiomyocyte viability, reduced apoptosis, inhibited NLRP3 inflammasome formation, and decreased secretion of inflammatory cytokines TNF‐α, IL‐1β, and IL‐6.^61^ miR‐495 ameliorates cardiac microvascular endothelial cell injury and inflammatory response by inhibiting the NLRP3 inflammasome signaling pathway.^62^ miRNAs may directly affect NLRP3 transcription by binding to the 3′UC region of NLRP3. Long‐chain noncoding RNA ZFAS1 indirectly regulates SESN2 by functioning as a competitive endogenous RNA (ceRNA) to alleviate sepsis‐induced cardiomyocyte damage.^63^

### 3.5 Calcium ion regulation model

Inflammatory cascade response can produce myocardial contractile disorders through calcium response.^64^, ^65^ As a second messenger, calcium ions also play an important role in oxidative stress, inflammatory response, and mitochondrial dysfunction that occur in SCM.^66^ Ca^2+^ activation and some metabolic changes are upstream signals of NLRP3 activation, and these pathways are interconnected with each other. It was found that cytoplasmic Ca^2+^ concentration increased in all cells under the action of different NLRP3 inflammasome activators. The Ca^2+^ signaling pathway is an important part of the NLRP3 inflammasome activation process. Inhibition of abnormal Ca^2+^ flow can reduce IL‐1β levels.^67^ Calcium‐sensitive receptors belong to a family of G‐protein‐coupled receptors, which activate NLRP3 inflammasome with the participation of phospholipase C, which catalyzes the production of inositol‐1,4, 5‐triphosphate and induces the release of Ca^2+^ stored in the endoplasmic reticulum (ER). Ca^2+^ in the cytoplasm promotes the assembly of inflammasome and stimulates calcium‐sensitive receptors to reduce the concentration of camp in the cell. To some extent, NLRP3 can be assembled smoothly by reducing the binding of the camp to NLRP3.^68^ Other related studies have shown that calcium‐sensitive receptor‐related family member 6A also has similar effects.^69^ Intracellular Ca^2+^ signaling pathway can trigger mitochondrial damage or intracellular Ca^2+^ release, and regulate the activation of NLRP3 inflammasome through calmodulin‐dependent protein kinase Ⅱ activation of transforming growth factor‐β activation of kinase 1/c‐Jun amino‐terminal kinase pathway.^70^ The NLRP3 activator induces extracellular Ca^2+^ influx, and the release of a large amount of Ca^2+^ from the ER Ca^2+^ reservoir into mitochondria can cause mitochondrial calcium overload. The outcome of this nourishment is mitochondrial damage, content release, and related molecular externalization, which in turn triggers the activation of NLRP3 inflammasomes.^71^ This suggests that intracellular Ca^2+^ signaling plays a crucial role in the activation of the NLRP3 inflammasome.

### 3.6 Endoplasmic reticulum stress

The ER is an important intracellular organelle that functions for protein folding, processing, and modification.^72^ Inflammation and endoplasmic reticulum stress (ERS) are closely related to its mechanism including REDOX regulating function obstacle, inflammation, overload, calcium homeostasis or protein expression, and so on.^73^ If the overaccumulation of unfolded proteins in the ER or the misfolded protein response (UPR) exceeds the load capacity, the normal physiological function will be destroyed, resulting in the imbalance of ER balance, and the occurrence of cell damage or apoptosis.^69^, ^74^ The misfolded protein response is the most important and widely studied pathway for ERS. In mammals, UPRs function using three ERS sensors: protein kinase‐like endoplasmic reticulum kinase (PERK), inositol‐dependent Kinase 1 (IRE1), and activated transcription factor 6 (ATF6). Studies have shown that the above three ERS sensors can activate the NLRP3 inflammasome through a complex mechanism to cause cell damage.^75^, ^76^ It was found that ERS activates PERK and IRE1, which activate NLRP3 inflammasome by promoting TXNIP expression.^77^ Activated IRE also activates NLRP3 inflammasome through the mitochondrial damage mechanism driven by Caspase‐2 and Bid.^78^

In addition, ER Ca^2+^ signaling promotes NLRP3 Inflammasome activation, while lipids activate NLRP3 inflammasome by triggering Ca^2+^ signaling or enhancing ER membrane fluidity, inducing the synthesis and release of inflammatory factors and affecting the development of inflammatory diseases.^79^ Studies based on animal models have shown that various drugs such as melatonin and liver X receptor agonists may attenuate sepsis‐induced myocardial dysfunction by inhibiting ERS.^80^, ^81^ Thus, ERS plays an important role in the activation of NLRP3 inflammasome. However, the precise molecular mechanism of ERS inducing SCM through activation of NLRP3 remains to be further studied and analyzed.

### 3.7 Pyroptosis

In recent years, pyroptosis has shown an exponential growth trend. It is a kind of programmed cell death which is different from apoptosis. Pyroptosis is a self‐protection method of cells against external damage, but its excessive activation will lead to body damage and sepsis.^82^ With the introduction of the concept of pyroptosis, we have gained further understanding and knowledge about SCM, and it was found that the appearance of cardiac dysfunction is also closely related to the occurrence of pyroptosis. The current mainstream view is that the pyroptosis mechanism is divided into the classical Caspase‐1‐dependent pathway and the nonclassical Caspase‐1‐dependent pathway. In the classical pathway, when the body recognizes PAMPs and DAMPs injury types under various endogenous and exogenous stimuli, the NLRP3 inflammasome is formed, which further participates in the activation of apoptosis protein or connector protein represented by connexin ASC. ASC can activate Caspase‐1 by collecting Pro‐Caspase‐1, and the activated Caspase‐1 can cause the release of IL‐1β, IL18, and other inflammatory factors and the cleavage of downstream target protein (gasderminD, GSDMD). The latter is cleaved into the C‐terminal domain and the N‐terminal domain by binding to phospholipid proteins on the cell membrane, resulting in the formation of cell membrane holes, cell rupture, and the release of inflammatory factors. The formation of such inflammatory factors and the initiation of cell rupture signaling pathways ultimately lead to cell death and peripheral inflammatory responses.^83^, ^84^, ^85^ IL‐1β and IL‐18 are the results of the joint action of the pyroptosis classical pathway and nonclassical pathway. Recent studies have shown that the NLRP3 inflammasome/Caspase‐1/IL‐1β pathway is involved in the occurrence of septic cardiac dysfunction caused by the excessive inflammatory response to a certain extent.^86^, ^87^ Reduced IL‐1β serum levels in septic Nlrp3 KO mice are accompanied by reduced cardiac and cardiomyocyte atrophy, improved cardiac diastolic and systolic function, and increased sepsis survival compared with septic WT mice. Inhibitors of the NLRP3 inflammasome (e.g., haemin, scutellarin, glyburide, and MCC950), IL‐1ß (anti‐IL‐1 antibody; anti‐IL‐1 Ab), IL‐1 RA, and NF‐κB (IKKβ‐directed NF‐κB inhibitor BMS‐345541), which are indicated, could be useful to prevent SCM.^28^ Inhibition of NLRP3 resulted in remission of LPS‐induced cardiac dysfunction in mice with sepsis and improved survival,^16^ This suggests that the protective effect of septic cardiac dysfunction can be accomplished by inhibiting NLRP3 inflammasome.^29^ Li showed that in LPS‐induced sepsis mice, STING‐IRF3 could activate the apoptosis and pyroptosis of cardiomyocytes by activating NLRP3 in mice, thus causing cardiac dysfunction.^88^ STING knockdown inhibited IRF3 phosphorylation and nuclear translocation, further attenuating NLRP3‐mediated cardiomyocyte inflammation, apoptosis, and neurotic sparing. Yang et al. found that TREM1 induced cardiomyocyte scorching by activating NLRP3 vesicles through the SMC4/NEMO pathway.^89^ Wu found that Intermedin1‐53 (IMD1‐53) inhibits NLRP3 activity in septic cardiomyocytes via the NLRP3/Caspase‐1/IL‐1β pathway and exerts a protective effect against SCM.^90^ This breakthrough point can provide a new direction for future prevention and treatment targets to reduce the risk of patients. Platelet activation.

### 3.8 Platelet activation

Numerous clinical data show that platelet activation is common in patients with sepsis complicated by cardiac dysfunction. Recently, it has been found that platelet and coagulation dysfunction may also play an important role in SCM.^91^ Platelets not only have a hemostatic effect, but they also become a major driver of innate and adaptive immune responses. The potential mechanism by which platelets may cause multiple organ damage is through activation of the NLRP3 inflammasome.^92^, ^93^, ^94^ NLRP3 signaling serves as a feedforward mechanism for platelet activation, and treatment with specific NLRP3 inhibitors not only attenuates NLRP3 activation in platelets but also reduces platelet activation stimulated by CLP and LPS in vitro. NLRP3 inhibition can affect platelet activation directly or indirectly. In vitro, studies have shown that IL‐1β stimulates platelet activation in an autocrine manner and that the antimicrobial peptides and inflammatory mediators released by activated platelets contribute to defense against infection.^95^ IL‐1β release from platelets was increased after LPS stimulation and significantly decreased after treatment with the NLRP3‐specific inhibitor MCC950. This suggests that the mechanism of platelet activation inhibition by MCC950 may be through a reduction in IL‐1β secretion.^94^ In addition, NLRP3 activation can occur in other immune cells, and these cells may indirectly activate platelets by releasing multiple factors.

## 4 SUMMARY

In summary, inflammasomes play a key role in the development and progression of innate immunity and immunoinflammatory diseases. It was found that NLRP3 inflammasomes are closely related to sepsis and SCM and can be involved in regulating SCM through cardiomyocyte inflammatory response and apoptosis, oxidative stress injury, calcium regulation, ERS, mitochondrial dysfunction, and exosomes. However, the mechanism of NLRP3 inflammasome assembly and activation remains to be further investigated. The role of NLRP3 inflammasome in myocardial dysfunction in sepsis deserves further exploration. The pathogenesis of SCM is complex, and the reliability of preventing multiorgan damage in sepsis by blocking the inflammasome pathway alone still requires more in‐depth studies, and there are still no global uniform diagnostic criteria for SCM that limit the further development of various studies in SCM. Researchers have developed some specific inhibitors for this pathway, offering new possibilities for the treatment of SCM. However, most of the current studies are animal experiments, and future benefit in humans still requires a large sample, multicenter, prospective clinical trials. In conclusion, the increasing research on the role of NLRP3 inflammasome in SCM may provide new ideas for the pathogenesis and treatment of SCM and improve patient prognosis.

## AUTHOR CONTRIBUTIONS

**Yuqi Wen**: Writing—original draft. **Yang Liu**: Writing—review and editing. **Weihong Liu**: Resources. **Wenli Liu**: Methodology. **Jinyan Dong**: Methodology. **Qingkuo Liu**: Visualization. **Hao Hao**: Funding acquisition. Hongsheng Ren Writing—review and editing.

## CONFLICT OF INTEREST STATEMENT

The authors declare no conflict of interest.
